# Infusing Mesenchymal Stromal Cells into Porcine Kidneys during Normothermic Machine Perfusion: Intact MSCs Can Be Traced and Localised to Glomeruli

**DOI:** 10.3390/ijms20143607

**Published:** 2019-07-23

**Authors:** Merel Pool, Tim Eertman, Jesus Sierra Parraga, Nils ’t Hart, Marieke Roemeling-van Rhijn, Marco Eijken, Bente Jespersen, Marlies Reinders, Martin Hoogduijn, Rutger Ploeg, Henri Leuvenink, Cyril Moers

**Affiliations:** 1 Department of Surgery—Organ Donation and Transplantation, University Medical Center, 9713 GZ Groningen, The Netherlands; 2 Department of Internal Medicine, Erasmus Medical Center, 3015 GD Rotterdam, The Netherlands; 3 Department of Pathology, University Medical Center, 9713 GZ Groningen, The Netherlands; 4 Department of Internal Medicine, University Medical Center, 9713 GZ Groningen, The Netherlands; 5 Institute of Clinical Medicine, Aarhus University, 8200 Aarhus, Denmark; 6 Department of Renal Medicine, Aarhus University Hospital, 8200 Aarhus, Denmark; 7 Department of Internal Medicine (Nephrology), Leiden University Medical Center, 2333 ZA Leiden, The Netherlands; 8 Oxford Transplant Centre, University of Oxford, OX3 7LJ Oxford, UK

**Keywords:** kidney transplantation, porcine, organ preservation, mesenchymal stromal cells, normothermic machine perfusion

## Abstract

Normothermic machine perfusion (NMP) of kidneys offers the opportunity to perform active interventions, such as the addition of mesenchymal stromal cells (MSCs), to an isolated organ prior to transplantation. The purpose of this study was to determine whether administering MSCs to kidneys during NMP is feasible, what the effect of NMP is on MSCs and whether intact MSCs are retained in the kidney and to which structures they home. Viable porcine kidneys were obtained from a slaughterhouse. Kidneys were machine perfused during 7 h at 37 °C. After 1 h of perfusion either 0, 10^5^, 10^6^ or 10^7^ human adipose tissue derived MSCs were added. Additional ex vivo perfusions were conducted with fluorescent pre-labelled bone-marrow derived MSCs to assess localisation and survival of MSCs during NMP. After NMP, intact MSCs were detected by immunohistochemistry in the lumen of glomerular capillaries, but only in the 10^7^ MSC group. The experiments with fluorescent pre-labelled MSCs showed that only a minority of glomeruli were positive for infused MSCs and most of these glomeruli contained multiple MSCs. Flow cytometry showed that the number of infused MSCs in the perfusion circuit steeply declined during NMP to approximately 10%. In conclusion, the number of circulating MSCs in the perfusate decreases rapidly in time and after NMP only a small portion of the MSCs are intact and these appear to be clustered in a minority of glomeruli.

## 1. Introduction

In an attempt to decrease waiting time for a donor kidney, donation after circulatory death (DCD) is starting to play a more prominent role in many transplant centres [1]. DCD is associated with an elevated risk of inferior early transplant outcome, as a result of the inevitable detrimental effect of warm ischaemia [2,3]. 

Preserving the function of ischaemically damaged kidney grafts is of vital importance for an effective transplantation. Pre-transplant machine perfusion could enable active organ conditioning and provides a platform for interventions prior to transplantation [4]. This is preferably done under near-physiological conditions, through (sub)normothermic machine perfusion at or just below 37 °C [5]. Normothermic machine perfusion (NMP) restores organ metabolism outside the body in absence of an allogeneic immune response prior to transplantation and this may reverse some effects of ischaemia. In addition, NMP may also allow for a better pre-transplant assessment of organ viability, compared to cold organ preservation techniques [6]. Finally, NMP could provide a platform for active interventions to an isolated organ prior to transplantation, such as administering cellular therapy.

Mesenchymal stromal cells (MSCs) could potentially improve the clinical outcome of kidney transplantations. MSCs are multipotent stromal cells that can be isolated from different tissue sources, including bone marrow and adipose tissue [7]. MSCs are defined by the following standard criteria: the potential to differentiate into osteoblasts, adipocytes and chondrocytes, adherence to plastic, the expression of surface markers CD105, CD90 and CD73, lack of expression of hematopoietic markers CD45, CD34 and endothelial marker CD31 [8,9]. MSCs have several attractive features, such as the modulation of innate immunity as well as adaptive immune responses [9,10]. In addition, MSCs are reported to facilitate repair of damaged tissue [10]. Current analyses suggest that treatment with MSCs will improve the outcome of cell and solid organ transplantation [11,12,13]. The desired immune modulating and regenerative effects can be achieved with either autologous or allogeneic MSCs [10]. So far, research has mainly focused on intravenous (IV) infusion of MSCs to kidney graft recipients after transplantation. These cells will most likely never reach the transplanted kidney, as studies show that IV infused MSCs do not migrate beyond the lungs [14]. In order for MSCs to be physically present in the injured target organ, direct infusion into the graft renal artery may be the solution. In addition, administering cellular therapy in a high concentration to only the isolated organ prior to transplantation could be advantageous, since the transplant recipient will not be systemically exposed to most of these cells and, in the absence of a host immune response, the effect of therapy may commence earlier. NMP could be an ideal platform for such an intervention [15]. The purpose of this study was to determine whether administering MSCs during NMP is technically feasible, what the effect of NMP is on the survival of MSCs, whether intact MSCs can be detected in the kidney after NMP and to which renal structures these cells localise.

## 2. Results

### 2.1. Normothermic Machine Perfusion

Three successful pilots were performed prior to the start of this study. After these pilots, no experiments were excluded or repeated. In total, 12 experiments were performed with A-MSCs, 10 with pre-labelled BM-MSCs and one experiment was performed with FeraTrack labelled MSCs in an MRI scanner. During normothermic machine perfusion all kidneys were functional, as they consumed oxygen and glucose and produced urine. The addition of MSCs did not lead to any macroscopic changes of the kidney nor to changes in haemodynamics.

### 2.2. Characterisation of MSCs Using Fluorescence-Activated Cell Sorting (FACS)

**Figure A1:**
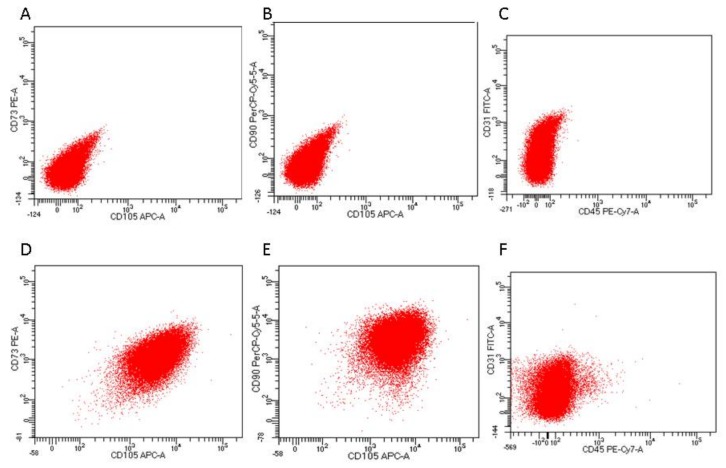
Characterisation of adipose tissue-derived MSCs. Flow cytometry analysis showed that unstained MSCs were negative for (**A**) CD73, (**B**) CD105, CD90, (**C**) CD31, and CD45. Stained MSCs were positive for (**D**) CD73, (**E**) CD105, CD90, and negative for (**F**) CD31, CD45.

FACS analysis showed that the analysed batch of passage 4 A-MSCs indeed expressed MSC markers CD105, CD73 and CD90 and lacked expression of lymphocyte marker CD45 and endothelial cell marker CD31 (Appendix A, Figure A1). The clinical grade BM-MSCs were provided by Leiden University Medical Center and were cultured and characterised at this GMP facility.

### 2.3. Renal Immunohistochemistry 

**Figure 1:**
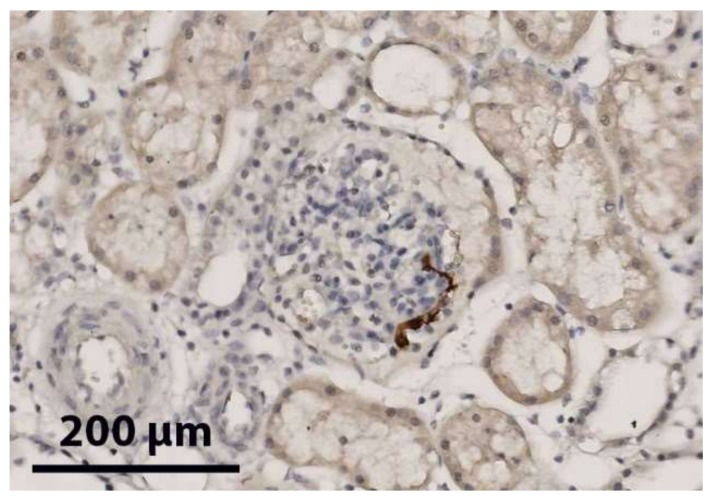
Red/brown immunohistochemical staining of MSCs in one glomerulus in a biopsy of the upper cortex of a kidney after NMP with 10^7^ MSCs added.

No MSCs were seen at t = 0 using immunohistochemistry, at the control slides, and neither in the 100,000 or 1 million MSC groups at all time points (*n* = 3 per group). In the 10 million group, in t = 360 min biopsies multiple MSCs could be identified (Figure 1). These MSCs were located in the lumen of glomerular capillaries. None were seen in the tubules. The presence of undamaged MSC nuclei in histological biopsies suggests that MSCs were still intact after tissue engraftment.

### 2.4. Fluorescence Microscopy in the Experiments with Pre-labelled MSCs

**Figure 2:**
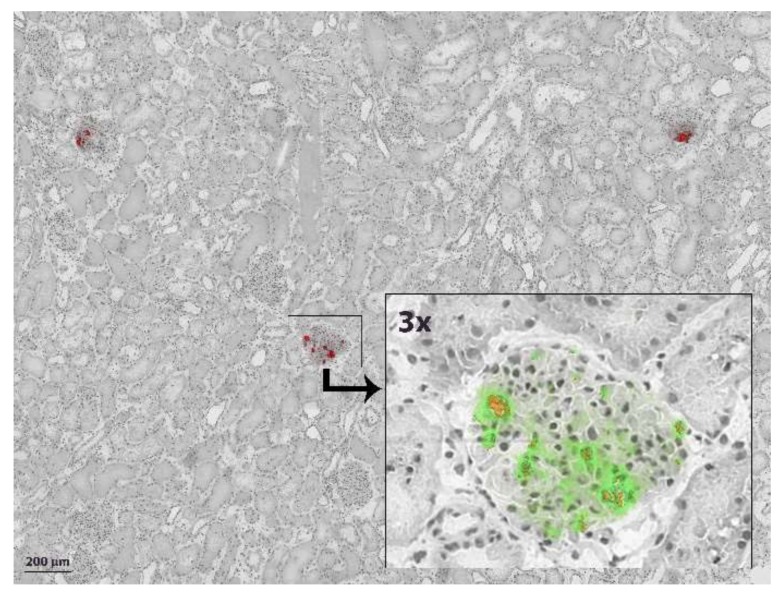
Glomerular PKH and Qtracker-625 fluorescence in cortical tissue after NMP with 10^7^ MSCs added. Both fluorescent channels combined (red) in overview, showing that most glomeruli were negative and only a minority of glomeruli were positive for infused pre-labelled MSCs. In addition, some positive glomeruli contained more than one MSC. Inset shows a 3x magnified confocal fluorescence microscopy image with emission wavelengths of both dyes separated: PKH = green, Qtracker-625 = orange.

For a more reliable detection, we decided to perform additional experiments in which MSCs were pre-labelled with Q-tracker 655 and PKH and infused in a perfusion system with and without a kidney (*n* = 5 per group). Selective detection of these fluorescent pre-labelled MSCs proved more straightforward compared to immunohistochemistry. Only a minority of glomeruli showed positive staining, however a striking finding was that fluorescent positive glomeruli often contained multiple MSCs, while most neighbouring glomeruli did not contain any MSCs at all. With confocal microscopy, the fluorescent emission wavelength of PKH26 (green) and Qtracker-655 (red) could be imaged separately. Most fluorescent hotspots were positive for both dyes. As PKH26 stains cell membranes and Qtracker-655 stains the cytoplasm, this finding indicates that these cells are likely to still be intact after NMP (Figure 2).

**Figure 3:**
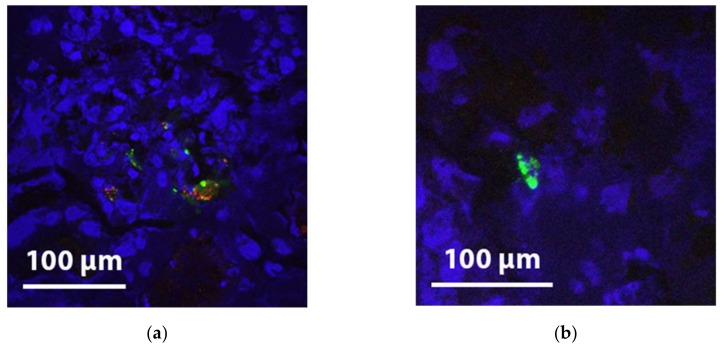
(**a**) Glomerular PKH (green) and Qtracker-625 (red), DAPI (blue) fluorescence in cortical tissue after NMP with 10^7^ MSCs added indicating intact MSCs; (**b**) fluorescent hot spot in the glomeruli, which solely has positive PKH staining and no Q-tracker signal. Such findings could indicate disintegrated MSCs.

We also consistently observed fluorescent hot spots in the glomeruli, which solely had positive PKH staining and no Q-tracker signal. As the intracellular Q-tracker label washes away, the outer membrane-bound PKH remains attached to membrane fragments when a cell breaks up. These hot spots most likely indicate disintegrated MSCs (Figure 3).

### 2.5. Detection of MSCs Using Flow Cytometry in the Experiments with Pre-labelled MSCs

**Figure 4:**
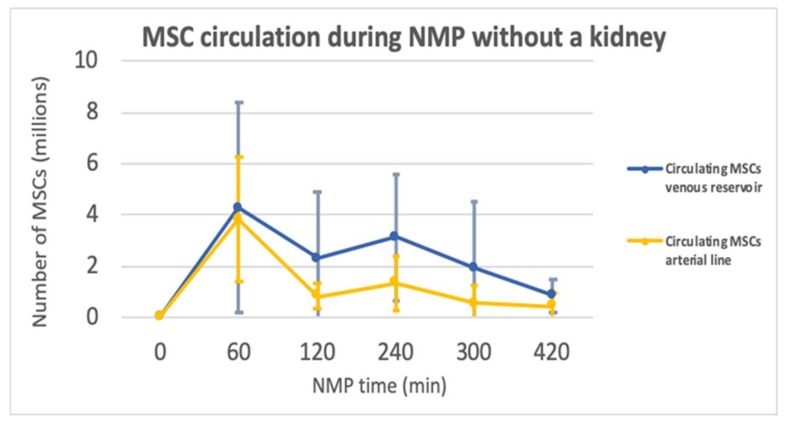
Graph of the MSCs circulating during NMP in the venous reservoir and the arterial line after the addition of 10 million MSCs at t = 60.

**Figure A2:**
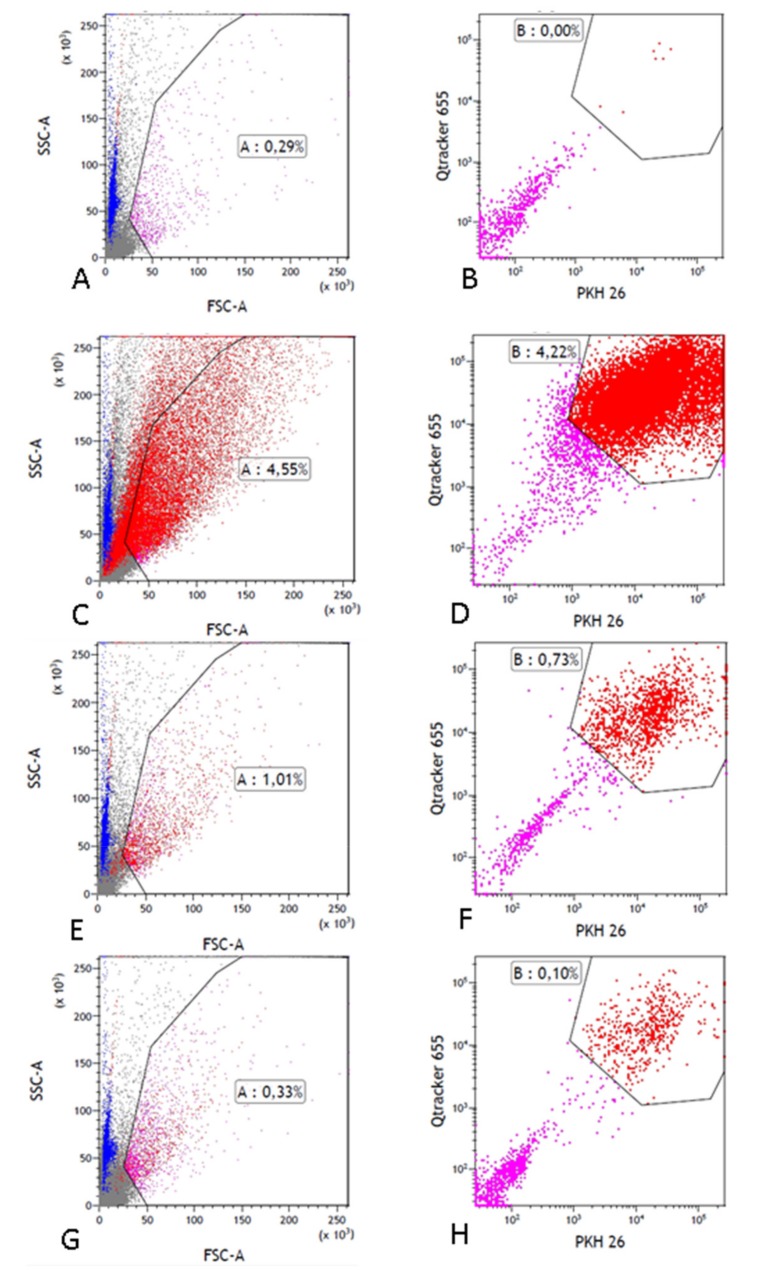
Example of flow cytometry results of perfusate reservoir samples. Cytograms of perfusate reservoir samples taken at different moments in time. Results shown in forward and side scatter cytograms and fluorescence signal strength cytograms of Qtracker655 and PKH26. (**A**) T = 0 sample moment, FSC-A, SSC-A cytogram, purple dots represent cells that fall within the MSC gating; 0.29% of all cells. (**B**) PKH26 and Qtracker655 cytogram of t = 0 sample, purple dots represent particles that fall in the auto fluorescence range. No dots visible in the MSC range gate. (**C**) T = 60 sample moment, FSC-A, SSC-A cytogram, purple dots represent cells that fall within the MSC gating; 4.55% of all cells. (**D**) PKH26 and Qtracker655 cytogram of t = 60 sample moment, purple dots represent particles that fall in the auto fluorescence range, red dots represent cells that fall within the MSC range gate; 4.22%. (**E**) T = 240 sample moment, FSC-A, SSC-A cytogram, purple dots represent cells that fall within the MSC gating; 1.01% of all the cells. (**F**) PKH26 and Qtracker655 cytogram of t = 240 sample moment, purple dots represent particles that fall in the auto fluorescence range, red dots represent cells that falls within the MSC range gate; 0.73%. (**G**) t = 420 sample moment, FSC-A, SSC-A cytogram, purple dots represent cells that fall within the MSC gating; 0.33% of all counted cells. (**H**) PKH26 and Qtracker655 cytogram of t = 420 sample moment, purple dots represent particles that fall in the auto fluorescence range, red dots represent cells that fall within MSC gate range; 0.10%.

Perfusate samples from the venous reservoir, as well as perfusate samples from the arterial line were analysed using flow cytometry to determine the isolated effect of machine perfusion on MSCs, while NMP was performed without a kidney in the circuit (*n* = 5). An example of the MSC detection using flow cytometry can be found in the Appendix A, Figure A2. Figure 4 shows the number of circulating MSCs at each given time point. Results are shown as mean ±SD. The MSCs were added at time point t = 60. The number of circulating MSCs in the reservoir initially dropped and then slightly increased again after a few hours of perfusion. After t = 240, the number of circulating MSCs in the venous reservoir decreased further. The yellow line, representing the number of circulating MSCs in the arterial line, shows similar results. Overall, during the perfusion the number of circulating MSCs seemed to be higher in the reservoir than in the arterial line. However, the number of cells circulating in the arterial line and in the venous reservoir were comparable at the end of each perfusion, and only approximately 10% of the number of cells that were initially infused were left.

**Figure A3:**
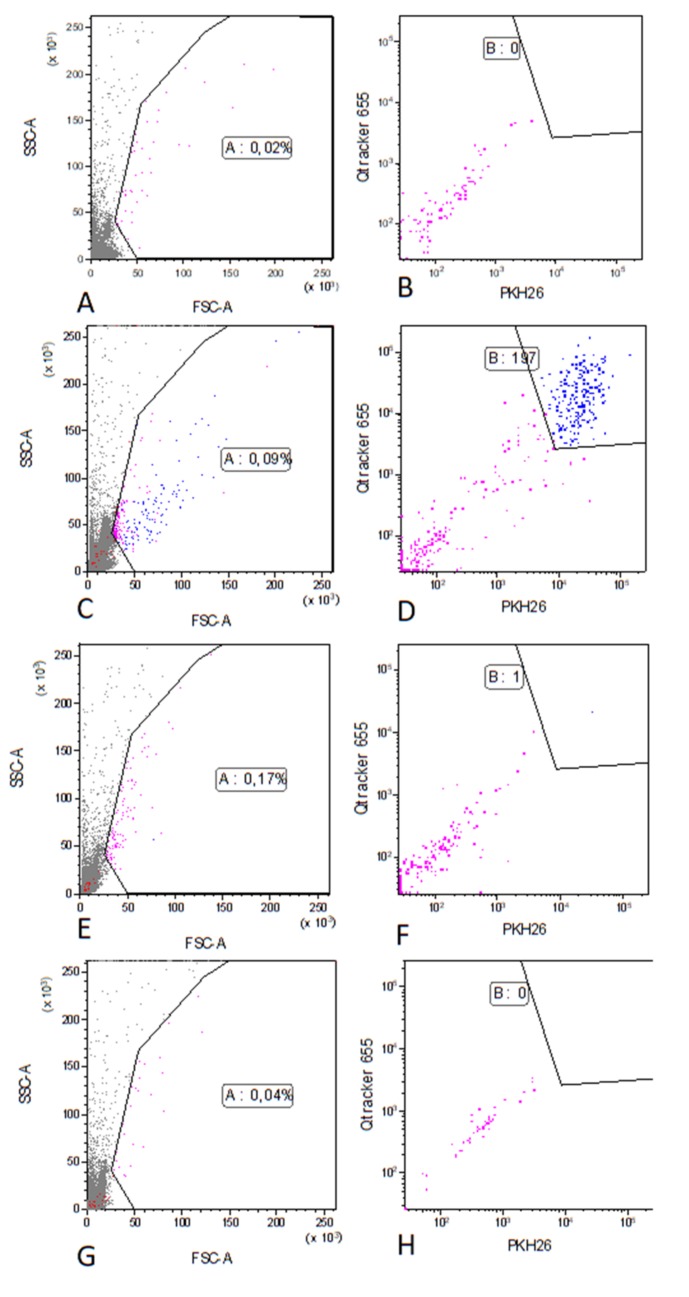
Example of flow cytometry results of reservoir samples. Results shown in forward and side scatter cytograms and fluorescence signal strength cytograms of Qtracker655 and PKH26. (**A**) T = 0 sample moment, FSC-A, SSC-A cytogram, purple dots represent cells that fall within the MSC gating; 0.02% of all cells. (**B**) PKH26 and Qtracker655 cytogram of t = 0 sample, purple dots represent particles that fall in the auto fluorescence range. No dots visible in the MSC range gate. (**C**) T = 60 sample moment, FSC-A, SSC-A cytogram, purple dots represent cells that fall within the MSC gating; 0.09% of all cells. (**D**) PKH26 and Qtracker655 cytogram of t = 60 sample moment, purple dots represent particles that fall in the auto fluorescence range, blue dots represent cells that fall within the MSC range gate; 0.22%. (**E**) T = 240 sample moment, FSC-A, SSC-A cytogram, purple dots represent cells that fall within the MSC gating; 0.17% of all the cells. (**F**) PKH26 and Qtracker655 cytogram of t = 240 sample moment, purple dots represent particles that fall in the auto fluorescence range, blue dot represents a cell that falls within the MSC range gate; 0.06%. (**G**) T = 420 sample moment, FSC-A, SSC-A cytogram, purple dots represent cells that fall within the MSC gating; 0.04% of all counted cells. (**H**) PKH26 and Qtracker655 cytogram of T = 420 sample moment, purple dots represent particles that fall in the auto fluorescence range, no dots visible in the MSC range gate.

**Figure A4:**
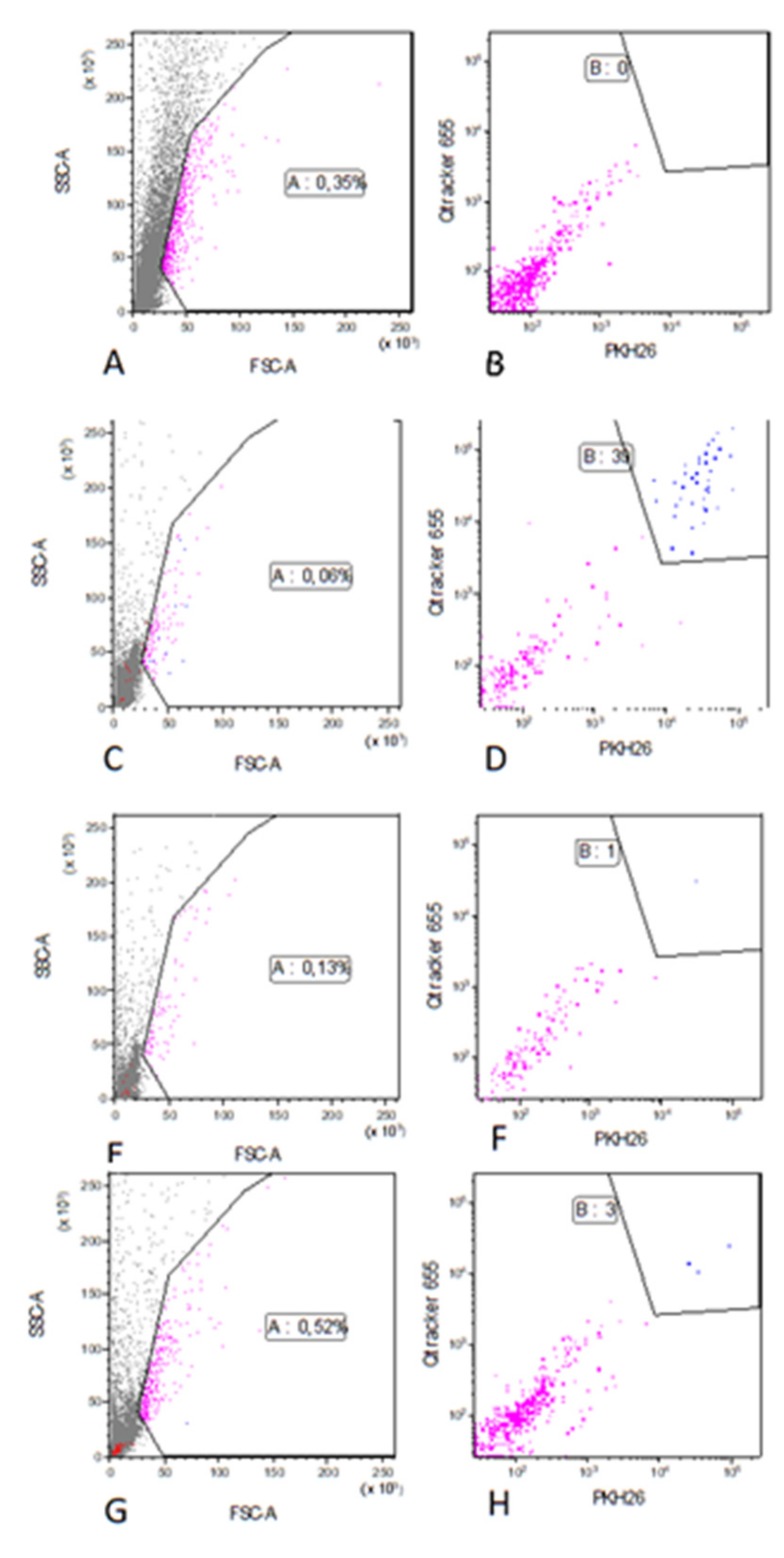
Example of flow cytometry results of arterial tubing samples. (A) T = 0 sample moment, FSC-A, SSC-A cytogram, purple dots represent cells that fall within the MSC gating; 0.35% of all counts. (B) PKH26 and Qtracker655 cytogram of T = 0 sample, purple dots represent particles that fall in the auto fluorescence range, no dots visible in the MSC range gate. (C) T = 60 sample moment, FSC-A, SSC-A cytogram, purple dots represent cells that fall within the MSC gating; 0.06% of all the cells. (D) PKH26 and Qtracker655 cytogram of T = 60 sample moment, purple dots represent particles that fall in the auto fluorescence range, blue dots represent cells that fall within the MSC range gate; 6.5% of the counted particles. (E) T = 240 sample moment, FSC-A, SSC-A cytogram, purple dots represent cells that fall within the MSC gating; 0.13% of all cells. (F) PKH26 and Qtracker655 cytogram of T = 240 sample moment, purple dots represent particles that fall in the auto fluorescence range, blue dot represents the cell that fall within the MSC range gate; 0.08% of the counted particles. (G) T = 420 sample moment, FSC-A, SSC-A cytogram, purple dots represent cells that fall within MSC gating; 0.52% of all counted cells. (H) PKH26 and Qtracker655 cytogram of T = 420 sample moment, purple dots represent particles that fall in the auto fluorescence range, the blue dots represent cells with a double fluorescent label that fall within the MSC gating; 0.06% of the counted particles.

In the perfusions with a kidney in the circuit (*n* = 5), kidney biopsies were dissociated into single-cell suspensions to be able to detect MSCs that are retained in the kidney. Perfusate samples from the arterial line, as well as from the reservoir and urine samples. were also analysed to detect the presence of MSCs. Results obtained by flow cytometry showed a very small number of double labelled counts in the enzymatically disrupted biopsies. Reservoir samples taken immediately after arterial MSC infusion showed a relatively high number of double labelled counts, which indicates that MSCs can travel through the kidney and are not fully retained at single pass. Reservoir samples taken at a later moment in time during the perfusion showed a lower level of circulating MSCs, indicating that the cells stop circulating over time. The arterial line perfusate samples showed very similar results (Appendix A, Figure A3 and Figure A4).

### 2.6. Detection of Iron Labelled MSCs during NMP in An MRI

**Figure 5:**
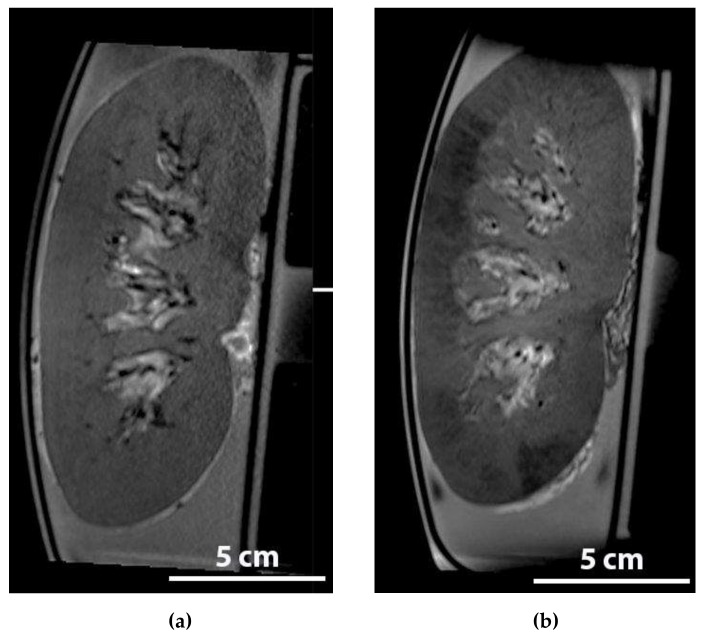
Anatomical, T2 weighted MRI sequence of a whole porcine kidney during normothermic ex vivo perfusion, in which water is typically displayed as white and iron (particles) will display as black. Examples of coronal reconstructions (**a**) baseline; (**b**) shortly after the infusion of 1 million FeraTrack labelled MSCs. The dark areas in the renal cortex represent the MSCs.

The T2 weighted scans generated during and shortly after the infusion of the labelled MSCs during NMP confirmed that the MSCs can indeed be traced to the renal cortex. The most striking finding was that in a well-perfused kidney the distribution of the MSCs was very inhomogeneous (Figure 5).

## 3. Discussion

To our knowledge, this is the first study in which human MSCs were administered during NMP of porcine kidneys. We added MSCs intra-arterially after 60 min of NMP and this did not lead to any changes in haemodynamics. On immunohistochemistry, it became apparent that after NMP most MSCs that were retained in the kidney were located inside the lumen of glomerular capillaries. These findings were confirmed with fluorescence microscopy of pre-labelled MSCs. As the capillary pores of glomeruli are significantly smaller than cultured MSCs it is quite plausible that the majority of cells remain inside the capillary lumen and do not migrate to other structures during perfusion [16,17,18]. Moreover, the relatively short NMP duration will most likely not allow for enough time to migrate to other structures. The finding that some glomeruli contained multiple fluorescent positive glomeruli, while most neighbouring glomeruli did not contain any at all, might be explained by anatomical heterogeneity in the microvasculature leading to MSCs following the path of least resistance during NMP. 

Although we were able to determine the number of circulating MSCs in a perfusion set-up without a kidney, we have not fully succeeded in determining the exact number of intact MSCs, which are located in the kidney after several hours of NMP, using flow cytometry. Reliably counting even several millions of infused MSCs among the billions of native renal cells proved to be quite challenging. Although microscopy clearly showed intact MSCs in renal tissue after NMP, we could not convincingly count large numbers of MSCs localised in the kidney with flow cytometry. This could be due to the fact that there were too many kidney cells in the enzymatically digested samples and too few total counts could be made to detect a substantial number of MSCs. The small number of positive counts could also be explained by the negative effect of the inevitable enzymatic digestion of kidney tissue into a single-cell suspension (to allow flow cytometry), which is performed using collagenase. Recent research has shown that collagenase has a significant impact on quality as well as quantity of intact MSCs [19].

We found that, in the perfusate, the number of MSCs decreased during NMP. As this was seen in the experiments with and without a kidney, it is probably the result of cells being exposed to pressure and flow during the perfusion itself. The fact that MSCs are tissue-adherent by nature, but are forced in a continuous non-physiological suspension during perfusion, might also play a role in the cell death [20]. After all, MSCs are tissue-residing stromal cells that may not be particularly adapted to the conditions inside blood vessels or perfusion tubing. As the cells decrease most rapidly during the final hours, shortening the perfusion time might result in better survival. Also, pre-conditioning, e.g. cytokine-activating, MSCs prior to ex vivo administration could be an interesting future refinement However, there is evidence that the beneficial effects of MSCs do not solely rely on their viability [21,22]. 

A possible limitation of our study could be that we used human MSCs in a porcine kidney. This might have led to a xeno-effect. However, this is relatively unlikely as our plasma and leukocyte-free NMP system did not incorporate an actual immune system and only a small number of tissue resident leukocytes may have been present during NMP. Moreover, research has shown that mesenchymal stem cells are well tolerated in an allogeneic or xeno-setting. Hence, when the kidney is transplanted and the pre-transplant infused MSCs come into contact with the host immune system, this should not lead to any negative effects [23,24,25,26,27].

This study did not focus on the effect of MSCs on renal function during NMP. This was a deliberate decision, as we feel it is of the utmost important to first increase our fundamental knowledge about the localisation and structural integrity of MSCs during NMP of a kidney. In addition, renal function is usually only very minimal during ex vivo organ perfusion and thus not a reliable marker for organ viability [28]. The best setting to test such functional outcome is in experiments that also incorporate actual transplantation. Before transplantation experiments are conducted, we feel that the next step should be to investigate cytokine secretion and post-NMP viability of infused MSCs. Hence, when we start to transplant MSC-treated kidneys we will have a better understanding of what effects can be expected. As it is our hypothesis that ischaemia-reperfusion injury could be mitigated, and because post-transplant renal function is superior as a result of MSC infusion during NMP, we are planning future porcine studies in which kidneys are actually transplanted to allow a much longer follow-up and functional assessment in vivo.

In conclusion, this study showed that it is possible to add cultured MSCs during NMP to an isolated porcine kidney in such a way that MSCs reach and reside in the kidney, with at least a small proportion of the administered cells remaining structurally intact and detectable. We were able to visualise MSCs in glomerular capillaries after their administration to the NMP circuit, but only when infused numbers were as high as 10^7^. Transplant studies are needed to determine whether targeting ischaemically damaged donor kidneys with MSCs is indeed beneficial for graft function and survival. The present study has provided the first important insights in survival and localisation of culture-expanded MSCs during NMP, and can be considered a first step in establishing machine perfusion as a platform to administer cellular therapy to damaged donor kidneys. Thus, an exciting new window of opportunity might emerge to actively pre-condition isolated organs in a fully controlled setting and in the absence of an immune response, before they are transplanted.

## 4. Materials and Methods

### 4.1. Organ and Blood Retrieval

In this pre-clinical study, we utilised porcine kidneys, as they closely resemble human kidneys in anatomical and physiological characteristics [29]. Kidneys and autologous, heparinised whole blood were obtained from two local slaughterhouses. Kidneys underwent 30 min of warm ischemia before cold flush with UW-CS (Belzer UW cold storage solution, Bridge to Life Ltd, Columbia, SC, USA) and storage on melting ice (0–4 °C). Washed, leukocyte depleted autologous red blood cell concentrate was obtained by filtering, centrifuging and washing porcine blood.

### 4.2. Perfusion Setup

**Figure 6:**
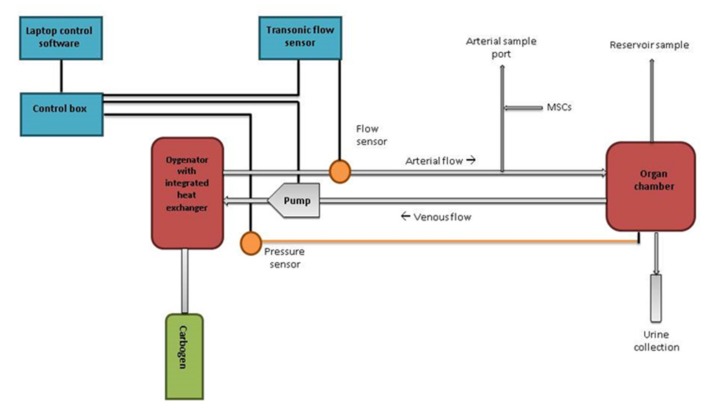
Schematic overview of the normothermic machine perfusion setup.

The perfusion circuit contained a pump unit (Medos Deltastream Pumpdrive DP2) with a centrifugal pump (both Medos Medizintechnik AG, Stolberg, Germany), an oxygenator/heat exchanger (Dideco Kids D100 neonatal oxygenator, Sorin LivaNova Nederland NV, Amsterdam, Netherlands, or Hilite 800 LT, Medos Medizintechnik AG, Stolberg, Germany) and a modified LifePort^®^ organ chamber with SealRing cannula (Organ Recovery Systems, Itasca, IL, USA). Perfusate temperature was kept at 37 °C. The perfusate was oxygenated with carbogen (95% O_2_/ 5% CO_2_). Flow was monitored using an ultrasonic clamp-on flow probe (Transonic Systems Inc., Ithaca, NY, USA). Pressure was measured directly at the SealRing cannula using a clinical-grade pressure sensor (TruWave disposable pressure transducer, Edwards Lifesciences, Irvine, CA, USA) (Figure 6). The setup was primed with 500 mL Williams’ Medium E (Gibco^®^ William’s Medium E + GlutaMAX™, Life Technologies Limited, Bleiswijk, Netherlands) supplemented with amoxicillin-clavulanate 1000 mg/200 mg (Sandoz B.V., Almere, Netherlands) and 40 gr of albumin (bovine serum albumin fraction V, GE Healthcare Bio-Sciences, Pasching, Austria) to obtain a physiological colloid concentration. After priming, 350 mL of pure red blood cells (RBCs) were added. The kidney was perfused in a pressure controlled, pulsatile sinusoid fashion at an arterial pressure of 120/80 mmHg during 7 h. Cold ischaemia time ranged from 3.5 to 5.0 h.

### 4.3. Isolation, Culture and Infusion of MSCs

Adipose tissue-derived MSCs were isolated from human perirenal fat, a clinical waste product of regular living donor nephrectomies in the University Medical Center Groningen. Informed consent was obtained from patients prior to organ donation. The choice to use human instead of porcine MSCs during this experiment was based on the idea that human MSCs would be easier to detect in the context of an abundance of porcine renal tissue. In addition, using human instead of porcine MSCs, means that we are testing the eventual clinical product. Due to the absence of circulating immune cells and antibodies, relevant xeno-effects are less likely to occur in an ex-vivo perfusion setup. The adipose tissue was mechanically disrupted and minced using a disposable scalpel knife. Next, the tissue was enzymatically digested using filtered 0.5 mg/mL collagenase type IV (Life Technologies), dissolved in RPMI medium (RPMI Medium 1640 + GlutaMAX™, Life Technologies) supplemented with 1% *p*/s (100 U/mL penicillin and 100 mg/mL streptomycin) during 30 min at 37 °C. After centrifugation at 700G for 7 min, the adipose tissue and medium were removed. The cell pellet was resuspended in minimal essential medium eagle-alpha (MEM-α) supplemented with 20% foetal bovine serum (FBS), 1% *p*/s and 2 mM L-glutamine (Life Technologies). The cells were then transferred to a cell culture flask and expanded at 37 °C. The culture medium was refreshed twice each week. The MSCs were trypsinised when they were80% to 90% confluent. For each experiment, an MSC suspension from freshly trypsinised MSCs was prepared 1 h before infusion. Reportedly, in humans, the most appropriate dose of MSCs is 0.4–10 x 10^6^ MSCs per kilogram body weight, as this does not lead to any significant adverse effects [30]. However, there is evidence that higher doses of MSCs are also safe [31]. As the weight of porcine kidneys we obtained was in the order of 250 g, we chose two doses that were within the earlier mentioned range and one that was well above. The correct number of cells (0, 10^5^, 10^6^ or 10^7^, *n* = 3 per group) were dissolved in 5 mL of Williams’ Medium E in a syringe. After 1 h of machine perfusion of the kidney, MSCs were added in a time span of 10 s via the arterial sample port, which was located very close to the renal artery. MSCs from passages 1 to 4 were used for these experiments. 

### 4.4. Pre-labelling of MSCs

As we found that MSC visualisation by means of immunohistochemistry in the first four experimental groups yielded sub-optimal discrimination, we decided to improve the detection of MSCs by pre-labelling cells with two strongly fluorescent dyes. We decided to switch to BM-MSCs for these experiments to confirm that these cells behave in the same manner. Also, these cells proved easier to culture than A-MSCs. For this purpose, 10 additional experiments were performed using 10^7^ bone-marrow derived pre-labelled MSCs. These BM-MSCs were provided by the Leiden University Medical Center (LUMC) and culture expanded by the Erasmus Medical Center Rotterdam. MSCs were double-labelled with PKH26 (Fluorescent Cell Linker kits, Sigma-Aldrich Chemie B.V., Zwijndrecht, Netherlands) and Qtracker 655^®^ (Cell Labelling kits, Thermo Fisher Scientific, Landsmeer, Netherlands) prior to infusion, following the protocol provided by the manufacturers. In five experiments, these pre-labelled BM-MSCs were infused during 7 h NMP of a porcine kidney, following the same protocol as mentioned above. In the other five experiments, pre-labelled BM-MSCs were infused into a circulating NMP circuit, without a kidney present, to assess the isolated effect of NMP on the survival of MSCs. In these perfusions, an artificial resistance was connected to the arterial outflow tubing to obtain similar haemodynamics during NMP as observed in experiments with a kidney present. 

### 4.5. Fluorescence-Activated Cell Sorting (FACS)

MSCs were immunophenotypically characterised by flow cytometry on a BD FACS Canto II (BD Biosciences). As the negative control we used 10^6^ unstained MSCs. Test samples contained 10^6^ MSCs with PE Mouse Anti-Human CD73, APC Mouse Anti-Human CD105, PerCP-Cy5.5 Mouse Anti-Human CD90, FITC Mouse Anti-Human CD31 and PerCP-Cy7 Mouse Anti-Human CD45 (all BD Biosciences). In addition, FACS analysis was used to determine the effect of NMP on the survival of MSCs during perfusion without a kidney with pre-labelled MSCs. For this purpose, hourly perfusate samples were taken. The MSCs could be identified by their fluorescence emission at two different wavelengths as a result of the PKH26 and Q-tracker 655 pre-labelling.

Flow cytometry was also performed on perfusate samples and enzymatically disrupted kidney biopsies from the experiments with pre-labelled MSCs with a kidney in the perfusion circuit. Of each sample, 1 million counts were obtained and analysed through flow cytometry.

### 4.6. Histology and Fluorescence Microscopy

A formalin fixed, paraffin embedded (FFPE) biopsy of the upper, lateral and lower renal cortex (T = 0; T = 180 and T = 360 min of NMP) was taken during the first 12 experiments. Visualisation of infused MSCs in the porcine renal tissue was performed at all three time points using a monoclonal mouse anti-human HLA Class I heavy chain (HC-10), rat anti-mouse polyclonal (RAMPO) and goat anti-rat polyclonal (GARPO) (all Sigma-Aldrich).

Cryopreserved cortical tissue biopsies (T = 420) of the additional experiments with pre-labelled MSCs were imaged with confocal microscopes: entire haematoxylin and eosin (HE)-stained slides were scanned in visual light to assess morphology (Leica SP8, Leica Microsystems, Wetzlar, Germany) and slides stained with mounting medium with 4,6-diamidino-2-phenylindole dihydrochloride (DAPI) (Vectashield mounting medium with DAPI, H-1200) (each only 4 µm apart from the corresponding HE-stained slide) were imaged at the emission wavelength corresponding to each of the fluorescent dyes, during excitation with the appropriate wavelength laser (LSM Zeiss 780 NLO, Carl Zeiss Microscopy, Jena, Germany). HE and fluorescence images were merged using Photoshop CC software (Adobe Systems Incorporated, San José, CA, USA).

### 4.7. Detection of Iron Labelled MSCs during NMP in An MRI

To visualise the distribution of MSCs in the kidney during NMP, 1 million BM-MSCs were labelled with FeraTrack Direct MRI contrast agent (Miltenyi Biotec, Leiden, the Netherlands). FeraTrack labels the MSCs with superparamagnetic iron oxide nanoparticles. During NMP in an MRI these labelled MSCs were infused whilst scanning in one porcine kidney.

## Abbreviations

DAPI	4,6-diamidino-2-phenylindole dihydrochloride
DCD	Donation after circulatory death
FACS	Fluorescence activated cell sorting
FBS	Foetal bovine serum
FFPE	Formalin-fixed paraffin-embedded
GARPO	Goat anti rat polyclonal
HE	Haematoxylin and eosin
MEM-α	Minimal essential medium eagle-alpha
MRI	Magnetic resonance imaging
MSCS	Mesenchymal stromal cells
NMP	Normothermic machine perfusion
RAMPO	Rat anti-mouse polyclonal
